# Changing surface ocean circulation caused the local demise of echinoid *Scaphechinus mirabilis* in Taiwan during the Pleistocene–Holocene transition

**DOI:** 10.1038/s41598-022-11920-3

**Published:** 2022-05-17

**Authors:** Sze Ling Ho, Jia-Kang Wang, Yu-Jou Lin, Ching-Ren Lin, Chen-Wei Lee, Chia-Hsin Hsu, Lo-Yu Chang, To-Hsiang Wu, Chien-Chia Tseng, Hsiao-Jou Wu, Cédric M. John, Tatsuo Oji, Tsung-Kwei Liu, Wen-Shan Chen, Peter Li, Jiann-Neng Fang, Jih-Pai Lin

**Affiliations:** 1 Institute of Oceanography, National Taiwan University, Taipei, Taiwan; 2 Department of Geosciences, National Taiwan University, Taipei, Taiwan; 3 Institute of Earth Sciences, Academia Sinica, Taipei, Taiwan; 4 Imperial College London, Prince Consort Road, London, SW7 2BP UK; 5 University Museum, Nagoya University, Furo-cho, Nagoya, 464-8601 Japan; 6 Department of Earth Sciences, Tennessee Tech University, Cookeville, TN 38505 USA; 7 Collection Management Department, National Taiwan Museum, Taipei City, Taiwan

**Keywords:** Biochemistry, Ecology, Biogeochemistry, Climate sciences, Environmental sciences, Ocean sciences

## Abstract

Abundant fossil specimens of *Scaphechinus mirabilis*, now occurring mostly in temperate waters, have been found in the Toukoshan Formation (Pleistocene) in Miaoli County, Taiwan. Environmental changes leading to its extirpation (local extinction) have thus far been elusive. Here, we reconstruct past environmental and oceanic conditions off northwest Taiwan by analyzing clumped isotopes, as well as stable oxygen isotopes, of well-preserved fossil echinoid tests collected from the Toukoshan Formation. Radiocarbon dates suggest that these samples are from Marine Isotope Stage 3 (MIS 3). Paleotemperature estimates based on clumped isotopes indicate that fossil echinoids were living in oceanic conditions that range from 9 to 14 °C on average, comparable with the estimate derived for a modern sample from Mutsu Bay, Japan. Notably, this temperature range is ~ 10 °C colder than today’s conditions off northwest Taiwan. The substantially lower temperatures during ~ 30 ka (MIS 3) compared to the modern conditions might be due to the rerouting of surface currents off northwest Taiwan when the sea level was ~ 60 m lower than today, in addition to the cooling caused by a lower atmospheric CO_2_ level during the Last Glacial Period. Colder waters brought here by the China Coastal Current (CCC) and the existence of shallow subtidal zones termed “Miaoli Bay” (mainly located in the present-day Miaoli county) during MIS 3 plausibly sustained generations of *S. mirabilis*, yielding tens of thousands of fossil specimens in the well-preserved fossil beds. The likely extirpation driver is the drastic change from a temperate climate to much warmer conditions in the shallow sea during the Pleistocene–Holocene transition.

## Introduction

Taiwan is situated between the Eurasian Plate and the Philippine Sea Plate. The main island of Taiwan is divided into several tectonic zones from west to east, including the Coastal Plain, Western Foothills, Hsueshan Range, Backbone Range, and Coastal Range. Cenozoic and often fossiliferous strata with shallow marine deposits are commonly exposed on the western side (Coastal Plain and Western Foothills) of the island. In particular, the Toukoshan Formation in Miaoli County is one of the well-known fossil deposits rich in marine invertebrate faunas, including mollusks, echinoids, crabs, corals, bryozoans, ostracods, barnacles, foraminifers, and sponge spicules^1,2^. A total of 134 species of mollusks have been reported from a single locality^3^.

**Figure 1:**
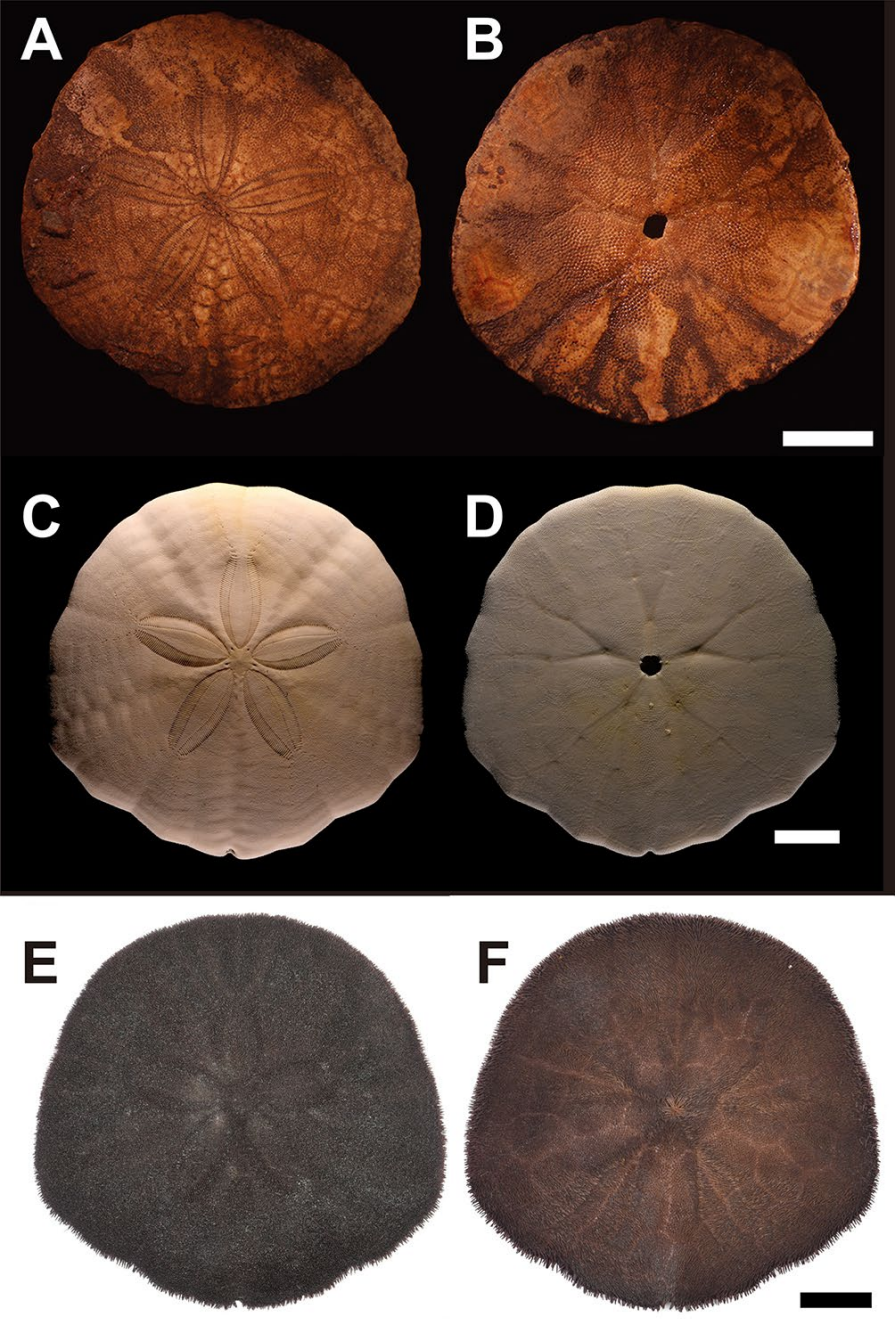
Fossils and living *Scaphechinus mirabilis* A. Agassiz, 1864. Scale bars = 10 mm. Aboral views (**A**,**C**,**E**); oral views (**B**,**D**,**F**). (**A**,**B**) Fossil specimens (C10), Pleistocene, Miaoli, Taiwan. (**C**,**D**) Denuded modern specimen (SM002) used for clumped isotope analyses (Fig. 3B). (**E**,**F**) Living specimen (SM100) with intact spines. Figure was created with CorelDRAW X7 Graphic (https://www.coreldraw.com/en/product/coreldraw/).

*Scaphechinus mirabilis* A. Agassiz, 1864 (Fig. 1) is the most abundant fossil echinoid species found in Taiwan with dense aggregations^4^. Over the years, more than 20,000 sand dollar specimens have been collected from the study area by amateurs and researchers, suggesting that the northwestern coast of Taiwan was once a favorable environment for *S. mirabilis* to thrive. More importantly, fossil assemblages here represent multiple generations and exhibit size variations, ranging from 9.5 to 62.4 mm in size^5^, allowing detailed studies on their ontogenetic changes^6,7^. Their living distributions at present, however, occur exclusively in cooler waters north of mainland Taiwan, such as northern China, Japan^8^, Korea, and the Russian Far East^9^. Based on the comparison with the modern analog, the default hypothesis for explaining the disappearance of *S. mirabilis* in Taiwan is climate change, but exactly what caused their extirpation in Taiwan remains a mystery due to a scarcity of Pleistocene temperature data from northern Taiwan.

**Figure 2:**
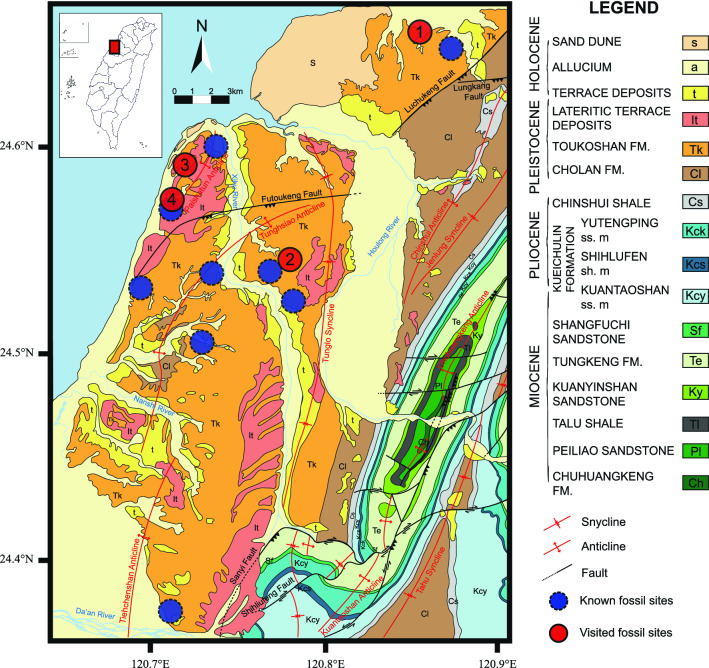
Geologic map and legends of stratigraphic units and major structures of studied area are generated based on 1:50,000 geologic maps (Paishatun, Miaoli, Tachia, and Tungshih) released by the Central Geological Survey of Taiwan with additions of fossil localities. Studied specimens are a subset of museum specimens deposited at the Department of Geosciences, National Taiwan University (NTUG) (Ref.^5^). Map was created with Adobe illustrator CS6 (https://www.adobe.com/products/illustrator.html).

Clumped isotope thermometry has gained popularity in the past decade, due in part to its independence from past seawater chemistry. It has been applied to biogenic carbonates, including foraminifers, corals, brachiopods, cephalopods, coccoliths, and echinoids^10^. Its application is generally limited by the large sample size requirement, which may preclude the measurement of small fossils such as planktic foraminifera widely used for paleoclimate reconstruction, but this is not an issue for the much larger test of sand dollars. Therefore, applying clumped isotope thermometry to the now extirpated sand dollars of *S. mirabilis* from Miaoli has the potential to reconstruct past environmental conditions wherein they thrived (Fig. 2). In addition, a few specimens of *S. mirabilis* were radiocarbon dated to further constrain the age of the samples retrieved from the fossil-bearing unit strata in the Toukoshan Formation (see refs.^11–13^). The lower part of the Toukoshan Formation consists of shallow marine sediments that have not undergone conspicuous diagenesis after burial; thus, the fossil sand dollars here are generally well preserved with almost no pore-filling cement around the tests.

## Results

### Stable oxygen isotopes

**Figure 3:**
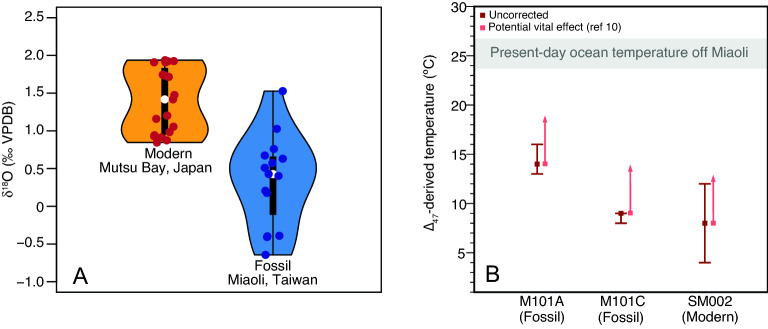
(**A**) Violin plots illustrating the distribution of stable oxygen isotopic compositions and (**B**) temperature estimates derived from clumped isotopes of both fossil and modern specimens of *S. mirabilis*. The error bars in panel B indicate the uncertainty of the ∆_47_ temperature for each sample, calculated from the standard error of the measurement (1 sigma, n = 3) and then propagated through the temperature calibration. Due to the non-linearity of ∆_47_-temperature calibration and the fact that the error estimate is rounded up to the nearest whole number after being converted to temperature unit, the error bars may be asymmetric (e.g. M101A) or one of the error limits may overlap with the mean value (e.g. M101C). See Table S2 for ∆_47_ data and Methodology section for more detail on the calculation. The red arrows in panel B indicate potential vital effect (0.0146 ± 0.0042‰) as reported by Davies and John^10^, corresponding to ~ 4.5 °C of underestimation. The gray shaded bar indicates the present-day ocean temperature averaged over the upper 50 m water column off Miaoli based on the climatology data retrieved from the closest grid point in the World Ocean Atlas 2018 (Ref.^14^)at 24.5° N 120.5° E and the Ocean Data Bank (http://www.odb.ntu.edu.tw/) at 24.5° N 120.75° E (details in Supplementary Information).

A total of 13 fossil samples selected from Site 2 in Miaoli (Fig. 2), Taiwan and 18 modern samples from Mutsu Bay, Japan were analyzed for stable oxygen isotopes. Each data point was generated from an aliquot of an intact specimen. For fossil samples, δ^18^O values range from − 0.6‰ to 1.5‰, with an average value of 0.3‰ ± 0.6‰. For modern samples, δ^18^O values range from 0.8 to 1.9‰, with an average value of 1.4‰ ± 0.4‰. The results indicate that the fossil samples show lighter δ^18^O values than those of the living samples from Japan (Fig. 3A).

### Clumped isotopes

Clumped isotope data were generated for two fossil samples from Miaoli (Site 2 in Fig. 2) and one modern sample from Mutsu Bay. ∆_47_-based temperature estimates (uncorrected for potential vital effect) for fossil samples from Miaoli are 9 °C (8–9 °C, 1SE) and 14 °C (13–16 °C, 1SE), respectively, while the modern sample from Mutsu Bay is 8 °C (4–12 °C, 1SE) (Fig. 3B).

### AMS radiocarbon dates

Two out of three samples (Site 2 in Fig. 2) yield dates that can be converted into calendar ages, while the last one was below the detection limit. They are 31,420 ± 280 and 48,620 ± 965 year BP, belonging to Marine Isotope Stage (MIS) 3 (Table 1), in agreement with published data^12^ based on mollusk shells from the same unit. In addition, one modern sample (SM003) from Japan was also analyzed; the datum suggests a modern age for the sample.

**Table 1:** Comparisons of ^14^C dates based on modern (SM003) and fossil (M22-M24) sand dollars and fossil mollusks reported in Peng et al.^12^.

Lab code	Sample ID	Species	pMC (%)	∆14C (%)	14C age (yr BP)	Calendric age (yr BP)	MIS	Sources
NTUAMS-6682–1	SM003	*S. mirabilis*	107.84 ± 0.84	78.4 ± 0.6	Modern	Modern		This study
NTUAMS-7067–1	M22	*S. mirabilis*	3.21 ± 0.08	− 967.9 ± 24.3	27,623 ± 202	31,420 ± 280	3	This study
NTUAMS-7068–1	M23	*S. mirabilis*	0.36 ± 0.02	− 996.4 ± 51.3	45,211 ± 413	48,620 ± 965	3	This study
NTUAMS-7069–1	M24	*S. mirabilis*	< 0.11	< − 998.9	> 55,000			This study
	α	*Pecten byoritsuensis*				44,900 ± 1400	3	Ref.^12^
	β	*Pecten byoritsuensis*				36,700 ± 600	3	Ref.^12^
	γ	*Pecten byoritsuensis*				30,900 ± 300	3	Ref.^12^

*MIS* Marine Isotope Stages. Modern is younger than AD 1950.

### X-ray diffraction

**Figure 4:**
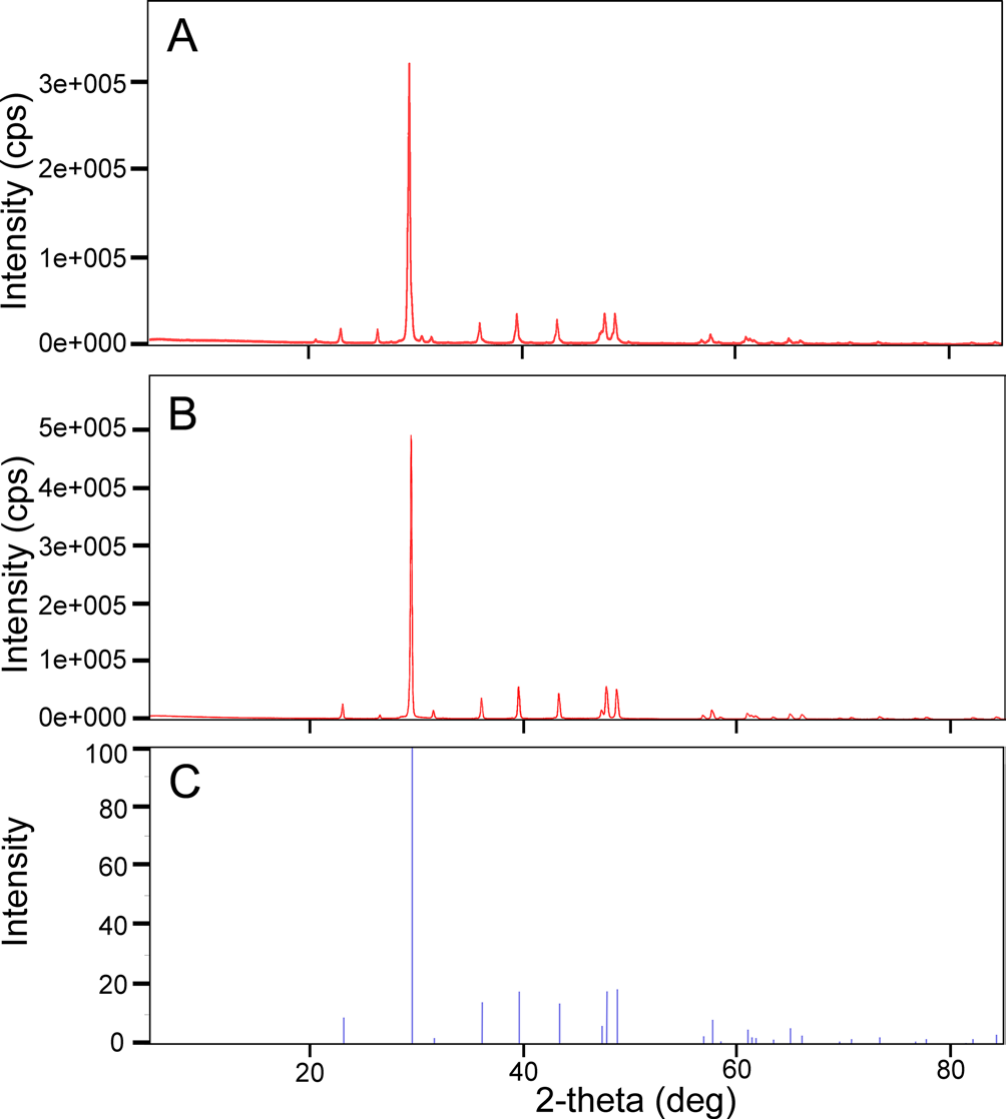
X-ray diffraction analyses of fossil (**A**) and modern (**B**) sand dollar *S. mirabilis* compared with the standard spectrum of high Mg calcite (**C**). Figure was edited and the line thickness was enhanced with Adobe illustrator CS6 (https://www.adobe.com/products/illustrator.html).

In order to assess the preservation quality of fossil specimen, both fossil and modern samples were analyzed. The dominant mineral spectra in both fossil and modern samples match with the Mg-rich calcite standard (Fig. 4). Fossil sample (SM2001) has some minor impurities, such as graphite, that is not present in the modern sample (SM2003).

## Discussion

The fossil sand dollars were retrieved from the Toukoshan Formation and are generally thought to be Pleistocene in age^11,13,15^. Radiocarbon dates of intact *Pecten* shells from the same strata suggest an age range of 30–50 ka (Peng et al.^12^) (Table 1). This age range is in agreement with the radiocarbon dates of our sand dollar samples. Among the three sand dollar samples sent for AMS radiocarbon analyses, two yield data that can be converted into calendar ages of 31,420 ± 280 and 48,620 ± 965 year BP. These ages correspond to Marine Isotope Stage 3 when the global mean sea level was 25 to 87 m lower than the present day^16^. Meanwhile, the radiocarbon content of one of the samples is below the detection limit, suggesting that it is older than 50 ka. Thus, our results based on sand dollars in part corroborate previous radiocarbon dates based on mollusk shells (Table 1). Combining our new radiocarbon dates with findings from previous studies^11,13,15^, we postulate that the fossil-bearing strata occurred during the late Pleistocene. The youngest dated specimen corresponds to ~ 30 ka BP (MIS 3 of the Last Glacial), which may mark the last occurrence of the species at this locality.

Stable oxygen isotope values of biogenic carbonate reflect the oceanic conditions at the time of biomineralization. Sand dollar specimens from the Toukoshan Formation are generally well preserved, with little compaction in the internal stereom, and the interconnecting pores still retain their original geometry; at least three types of stereoms are still recognizable^5^. Because more than a thousand intact and incomplete tests were available for this study, only the best-preserved samples were analyzed (see more detail on preservation state and selection criteria in “Materials and Methods” section). These samples show minimal effects of diagenesis and are thus useful as recorders of past environmental changes in their habitat. The modern and fossil sample groups show different δ^18^O distributions (Fig. 3A). The δ^18^O values of modern samples range between 0.8 and 1.9‰, whereas those of the fossil samples range between − 0.6 and 1.5‰. With the exception of two samples, the δ^18^O values of the fossil samples are lower than those of the modern samples, on average by ~ 1‰ (based on the median value of distribution; white circles in Fig. 3A). The δ^18^O difference between the two groups is likely due to the combined effect of seawater δ^18^O and temperature. Although modern-day seawater δ^18^O values in the Taiwan Strait and Mutsu Bay are comparable within 0.1‰ according to the global seawater δ^18^O gridded product^17^, the seawater δ^18^O values off northwest Taiwan (present-day Taiwan Strait) might have been quite different during the Last Glacial Period as a result of changes in the hydrological cycle. Global mean seawater δ^18^O was lower by ~ 0.5‰ during MIS 3 relative to the Holocene^18^. Accounting for this effect further amplifies the δ^18^O difference between the modern and fossil sand dollar samples to ~ 1.5‰. However, it is unknown whether there has also been local seawater δ^18^O change off northwest Taiwan during MIS 3, thereby complicating the attribution of calcite δ^18^O changes to the effects of seawater δ^18^O and/or temperature. Temperature changes in the growth environment can alter the δ^18^O signature of biogenic carbonate by an approximately 0.23‰ decrease with a 1 °C temperature increase^19^. The δ^18^O values of the fossil samples from Miaoli are on average ~ 1.5‰ (including global ice volume effect) lower than those of the modern samples from Mutsu Bay, suggesting that the waters off northwest Taiwan might have been ~ 6.5 °C lower than those in present-day Mutsu Bay. However, the robustness of the aforementioned temperature estimate is hampered by uncertainties associated with local seawater δ^18^O changes during MIS 3 and strong vital effects in the δ^18^O of the echinoid test^20^.

Unlike δ^18^O, carbonate clumped isotope (∆_47_) thermometry is independent of seawater chemistry^21^, and clumped isotope composition in echinoid tests is homogenous interskeletally^10^, rendering it a suitable tool to reconstruct past seawater temperature when *S. mirabilis* thrived in Taiwanese waters off Miaoli. The ∆_47_ value of a living specimen of *S. mirabilis* from Mutsu Bay^22^ yields a temperature estimate of 4–12 °C (mean value of 8 °C), matching that obtained from a recent seafloor temperature survey^23^. We note that a recent study on modern echinoids shows a positive offset in the echinoid ∆_47_ value relative to the ∆_47_-temperature relationship in inorganic calcite (0.0146 ± 0.0042‰)^10^, corresponding to an approximately 4.5 °C underestimation of the precipitation temperature at 15 °C. This offset is consistent among echinoid groups and thus may also affect *S. mirabilis* even though this species was not examined in the aforementioned calibration study. Accounting for this offset would slightly increase the ∆_47_-temperature of our sample (from ~ 8 to ~ 12 °C for the Mutsu Bay sample; Fig. 3B), suggesting a slightly warmer habitat that is still within the range of modern distribution of *S. mirabilis*. This demonstrates the applicability of ∆_47_ thermometry to the *S. mirabilis* test.

Temperature estimates for the fossil samples from Miaoli range from 9 to 14 °C (not accounting for potential vital effect), agreeing within uncertainty with the ∆_47_-temperature estimate derived from the modern specimen in Mutsu Bay (uncorrected mean = 8 °C, 4–12 °C (1SE); Fig. 3B). This agreement implies that *S. mirabilis* off Miaoli thrived in an environment that is similar to the temperate waters of present-day Mutsu Bay in Japan. In other words, the species likely has not substantially adjusted their thermal habitat niche in the past tens of thousands of years. Notably, such an environment is substantially colder (by > 10 °C; > 6 °C if accounting for potential vital effect) than the coastal waters off Miaoli in the present-day Taiwan Strait (Fig. 3B; ~ 24 °C to 26 °C in the upper 50 m).

**Figure 5:**
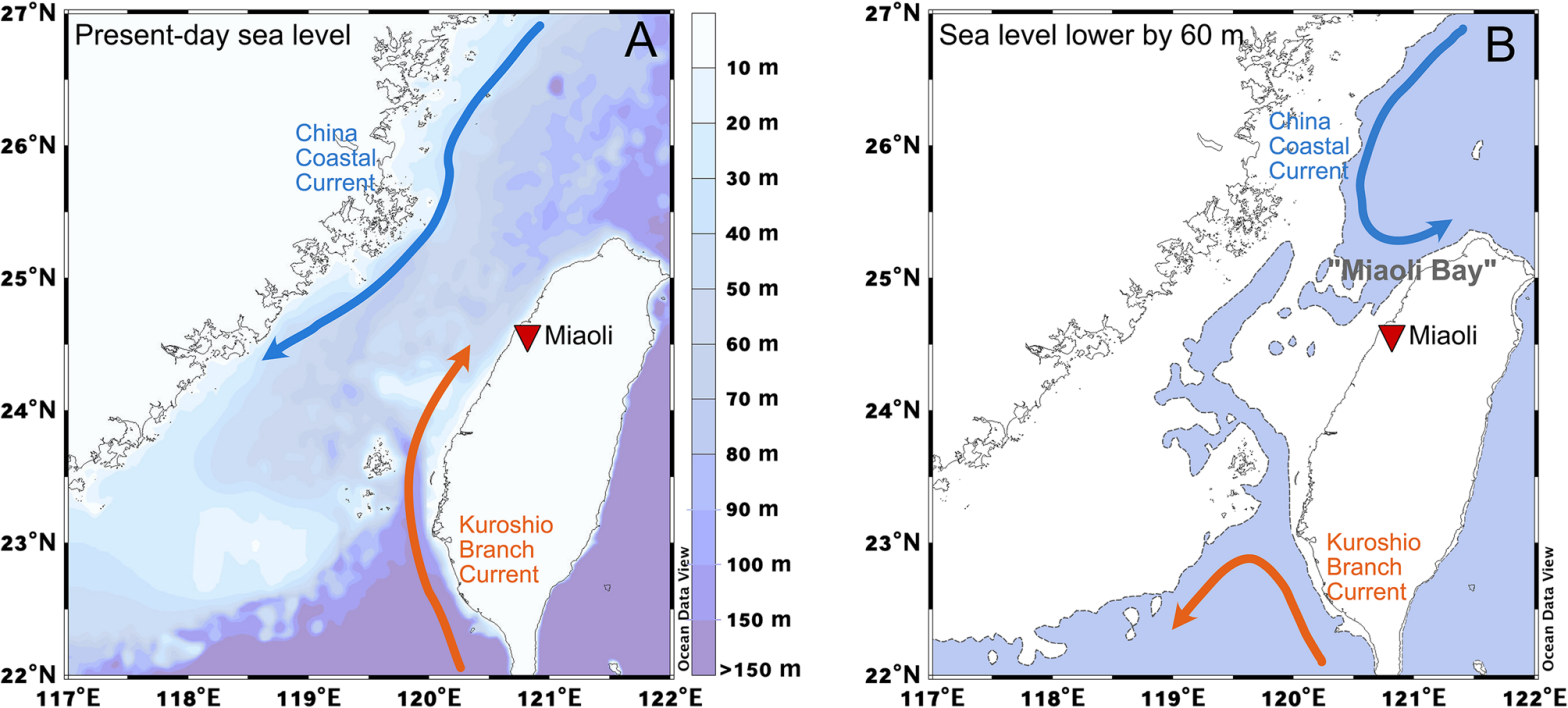
Surface ocean circulation in the Taiwan Strait at different sea levels. (**A**) Present-day sea level and (**B**) when the sea level was lower by ~ 60 m during MIS 3 (30–50 ka) (Ref.^16^). Color bar in panel A represents ocean bathymetry. In panel B, the area covered by ocean is shaded blue, while land is shaded white. Maps were generated using Ocean Data View (https://odv.awi.de/, version 5.5.1).

To the best of our knowledge, this is the first direct evidence of temperate waters off northwest Taiwan (in the present-day region of Taiwan Strait) where the subtidal zone was cool enough for temperate-to-cool water echinoid fauna to thrive during the Last Glacial Period. There are no existing marine temperature records for MIS 3 in the study area for comparison with our echinoid ∆_47_ temperatures. However, we note that glacial cooling of > 10 °C (> 6 °C if accounting for potential vital effect) here is substantially stronger than the ~ 3 °C cooling during the Last Glacial Maximum recorded in the southern Okinawa Trough off northeast Taiwan^24^. Unlike the eastern coast of Taiwan, which is under the direct influence of the intense Kuroshio Current that brings a large amount of heat from lower latitudes, the northwestern coast adjacent to the Taiwan Strait is influenced by both the warm Kuroshio Branch Current (seasonal range of 21–25 °C^25^) and cold China Coastal Current (CCC; 10–16 °C in winter^26^)(Ref.^27^). The flow path of CCC varies seasonally, and its transport to the west coast of Taiwan increases during winter (relative to summer). During MIS 3, when the sea level was ~ 60 m lower than today^16,28^, a large part of the present-day Taiwan Strait (Fig. 5A) was likely above sea level, forming a “land bridge” between Taiwan and mainland China (Fig. 5B). Such a “land bridge” would reroute the southward flow of CCC toward Taiwan, forming a bay (in present-day Miaoli County) “Miaoli Bay” that is habitable for *S. mirabilis* and cutting off the northward transport of the warm Kuroshio Branch Current. Under this circumstance, it is plausible that the influence of cold CCC during MIS 3 in our study area was substantially stronger than its counterpart today. This change in surface circulation, coupled with glacial cooling associated with the lower *p*CO_2_ during the Last Glacial, renders coastal waters colder by more than 6 °C possible on the northwestern coast of subtropical Taiwan.

“Land bridge” formation and the change in CCC dynamics in the Pleistocene might also contribute to the discrepancy between δ^18^O values of modern-day Mutsu Bay and Pleistocene “Miaoli Bay” (Fig. 3A). CCC has lower salinity than the surrounding residing shore water around western Taiwan^26^, and salinity has been known to be negatively correlated with δ^18^O in previous studies^29,30^. Therefore, the stronger influence of CCC in the Pleistocene might both contribute to the colder water and lower salinity, causing the δ^18^O to be lower than that of modern-day Mutsu Bay (Fig. 3A).

Lower ocean temperatures aside, the lower sea level during MIS 3 means that a broad subtidal zone in “Miaoli Bay” had the potential to sustain generations of *S. mirabilis*. This hypothesis explains for the first time the mystery, i.e., why fossil occurrences of *S. mirabilis* are exclusive within western Miaoli County and are absent in the southern portion of Taiwan, where the influence of the Kuroshio Branch Current would prevail despite the presence of the “land bridge”(Fig. 5B). Based on the fossil distribution on land (Fig. 2), Pleistocene “Miaoli Bay” may represent a shallow bay where *S. mirabilis* are abundant.

Other supporting evidence for the temperate climate in northwest Taiwan during the late Pleistocene comes from independent studies of coeval faunas, both marine and terrestrial in origin. Chen^31^ first reported the occurrence of cold-water molluscan species present in fossil assemblages from the same unit (e.g., site 3 in Fig. 2). He noted that while most species inhabit the latitudinal range of 20° to 25° N, certain species, including *Balcis candida Olivella spretoides*, *Terebra evoluta* (= *Duplicaria evoluta*), *Anadara tricenicosta* and *Paphia euglypta*, are typically restricted at present to waters further north in 35° to 40° N latitude. Thus, he hypothesized either that cool climate conditions prevailed or the influence of a cold ocean current in the studied region. Based on palynology studies of Taiwan, Liew^15^ identified intervals of Zones *Pinaceae* I, subzones H, F and I of the *Castanopsis* Zone, and Zones W2 and T2, all of which represent cool-climate stages during the Pleistocene. Among the Taiwanese megafaunas reported in Chen and Hsu^32^, the Taiwan Strait fauna, including *Palaeoloxodon naumanni penghunensis* and *Bubalus teilhardi*, represent the best examples of extinct mammals that once lived in Taiwan during the Ice Age.

All of the cold-climate mammalian species mentioned above are now extinct in Taiwan. It is estimated that at least one million individuals of *S. mirabilis* once lived in the shallow subtidal areas in western Taiwan for generations^4–7^. While they have been found in dense aggregates in the Toukoshan Formation, no living specimen near mainland Taiwan has been reported. Thus, its abrupt disappearance since MIS 3 represents a mass extirpation event. While Quaternary megafauna extinctions could be induced by human activities, such as hunting^33–39^, this is a clear case of a climate-driven demise because sand dollars have very little edible soft tissues that could be consumed by neither natural predators nor human beings. ∆_47_-based temperature estimates derived from the calcite test of now locally disappeared *S. mirabilis* provide unequivocal evidence that during MIS 3, the coastal waters of northwestern Taiwan were 9–14 °C (13–18 °C if accounting for potential vital effect), i.e., ~ 10 °C colder than present day (24–27 °C; Fig. 3B and Electronic Supplementary Material). Laboratory experiments demonstrate that seawater temperature exerts a strong influence on the survival rate of the larvae of *S. mirabilis*, which drops drastically to 0% when the seawater temperature is raised from 14 to 25 °C^9^. Therefore, we argue that the most likely driver for the extirpation of *S. mirabilis* in Taiwan is the physiological stress induced by increasing seawater temperature and the loss of habitat during the Pleistocene–Holocene transition.

## Materials and methods

### Sample collection and selection

Living specimens from Japan were retrieved from Mutsu Bay courtesy of Satoshi Takeda (Tohoku University). Fossil samples were collected from 4 localities within the Toukoshan Formation (Fig. 2 and Fig. S1). Samples are Pleistocene in age, thus, fossils are relatively young geologically and underwent minimal deep diagenetic alterations. To assess the preservation state of the samples, we further powdered both fossil and modern samples for X-ray diffraction (XRD; methods described below) analyses. The presence of the metastable high-magnesium calcite (HMC) in analyzed samples (Fig. 4) indicates the original composition of echinoderm ossicles and confirms our hypothesis. Out of > 1000 specimens collected, we selected only the best-preserved specimens (Site 2 in Fig. 2) for radiocarbon dating and geochemical analyses. Selections of samples are based on the following criteria: (1) preservation of stereom (Ref.^5^); (2) no obvious dissolution or organic staining; (3) no deformation and structural features; and (4) avoiding fast growing regions, including lantern and marginal plates, in order to prevent vital effect (Ref.^10^).

### X-ray diffraction (XRD) analysis

Samples were rinsed in deionized water, pulverized, and passed through a metal sieve of 500 µm pore size before XRD analysis. X-ray analysis was performed using a Rigaku Ultima IV diffractometer. A normal line focus CuK_a_ radiation tube was used with power settings of 40 kV, 44 mA, 1.76kw. Measurements were made using a D/teX Ultra High-Speed Detector with the detector discriminator set in a fluorescent reduction mode. All samples for a given processing condition were loaded into an automatic rotary sample changer, and diffraction patterns were recorded using a scan speed of 2.00 degrees/minute and a scan range from 5 to 85° θ/2θ. The raw data was imported into the PDXL software package for phase analysis and compared with the standard patterns (e.g., Fig. 4C) in the ICDD database (https://www.icdd.com/).

### Stable isotope measurement

The stable oxygen and carbon isotopes of the samples (n = 34) were analyzed at the Stable Isotope Laboratory of the University of Tennessee at Knoxville (https://eps.utk.edu/research/facilities_isotope.php). Prior to analysis, carbonate samples were cleaned and ground into powder. Measurements were performed on a Thermo-Finnigan Delta + XL Mass Spectrometer.

### Clumped isotopes measurement

Clumped isotope measurements were carried out in the Qatar Stable Isotope Laboratory at Imperial College London (ICL) using a fully automated prototype IBEX (Imperial Batch Extraction) system. Both living and fossil specimens were rinsed and ultrasonicated in water and dried overnight before being ground into powder. The carbonate powder was subjected to oxygen plasma ashing^40^ to remove organic matter, which may interfere with the measurement. The measurement followed the methodology outlined in Adlan et al.^40^. Briefly, samples of calcite powder (~ 4 mg) were reacted with 105% orthophosphoric acid at 90 °C for 10 min to generate carbon dioxide (CO_2_) gas. Water, sulfur, and hydrocarbons were removed by passing the CO_2_ gas through water traps, silver wool and a Porapak-Q trap. The purified gas was then analyzed using a dual inlet Thermo MAT 253 isotope ratio mass spectrometer (Thermo Instrument, Bremen, Germany) with Faraday collectors for *m/z* 44–49. Raw ∆_47_, δ^18^O and δ^13^C values were corrected and calculated using the open-source community software Easotope^41^. Pressure baseline correction was performed following the methodology developed at ETH Zürich^42^. Clumped isotope values are reported with respect to ETH carbonate standards (ETH-1, ETH-2, ETH-3, ETH-4) and at an acidification temperature of 90 °C, i.e., the Intercarb Carbon Dioxide Reference Frame (I-CDES)^43^. ∆_47_ values were converted to temperature units using a new community calibration curve^44^. This calibration curve is reported on the I-CDES scale and includes the high-temperature standards analyzed at ICL; thus, it is considered the most suitable calibration for our ∆_47_ data. The ∆_47_-based temperature estimate for each sample was based on 3 replicate measurements (Supplementary data). The mean, maximum and minimum values of the ∆_47_-based temperature were derived from the mean of the measured ∆_47_ value and the standard error (1 sigma) of the measurement. The maximum and minimum values are indicated by the error bars in Fig. 3B. This uncertainty thus reflects the overall analytical error and the heterogeneity of the sample. Note that the mean value is not in the middle of the interval because the ∆_47_-temperature calibration is non-linear, such that ∆_47_ changes at higher temperatures result in a greater difference in temperature than at lower temperatures. We report ∆_47_ temperature estimates both with and without accounting for the echinoid vital effect of 0.0146 ± 0.0042‰ reported by Davies and John^10^.

### Radiocarbon dating

Accelerated mass spectrometry radiocarbon data were analyzed at NTUG. Sample preparation processes followed the protocols in Lee et al.^45^. Briefly, the samples were cleaned by ultrasonication in deionized water before being ground into powder. Approximately 12 mg of powder was reacted with 100% phosphoric acid to produce CO_2_ gas, which was then purified and graphitized. Radiocarbon ages were converted into calendar age using the Calpal program with the IntCal13 database^46^.

## Supplementary Information

Supplementary Information.
