# Supplementary material for: Changing surface ocean circulation caused the local demise of echinoid Scaphechinus mirabilis in Taiwan during the Pleistocene–Holocene transition
Source: Sci Rep. 2022 May 17;12:8204. doi: 10.1038/s41598-022-11920-3 (PMC9114427; doi:10.1038/s41598-022-11920-3)
Supplement: Supplementary file 1 — Supplementary Information. [file 41598_2022_11920_MOESM1_ESM.pdf]

Changing surface ocean circulation caused the local demise of echinoid *Scaphechinus mirabilis* in Taiwan during the Pleistocene-Holocene transition

Sze Ling Ho[1], Jia-Kang Wang[2], Yu-Jou Lin[2], Ching-Ren Lin[3], Chen-Wei Lee[2], Chia-Hsin Hsu[2], Lo-Yu Chang[2], To-Hsiang Wu[2], Chien-Chia Tseng[2], Hsiao-Jou Wu[2], Cédric M. John[4], Tatsuo Oji[5], Tsung-Kwei Liu[2], Wen-Shan Chen[2], Peter Li[6], Jiann-Neng Fang[7], and Jih-Pai Lin[2*]

[1]Institute of Oceanography, National Taiwan University, Taipei, Taiwan

[2]Department of Geosciences, National Taiwan University, Taipei, Taiwan

[3]Institute of Earth Sciences, Academia Sinica, Taipei, Taiwan

[4]Imperial College London, Prince Consort Road, London, SW7 2BP, U.K.

[5]University Museum, Nagoya University, Furo-cho, Nagoya 464-8601, Japan

[6]Department of Earth Sciences, Tennessee Tech, Cookeville, TN 38505, USA

[7]Collection Management Department, National Taiwan Museum, Taipei City, Taiwan

*Corresponding author Email: alexjplin@ntu.edu.tw

Electronic Supplementary Material

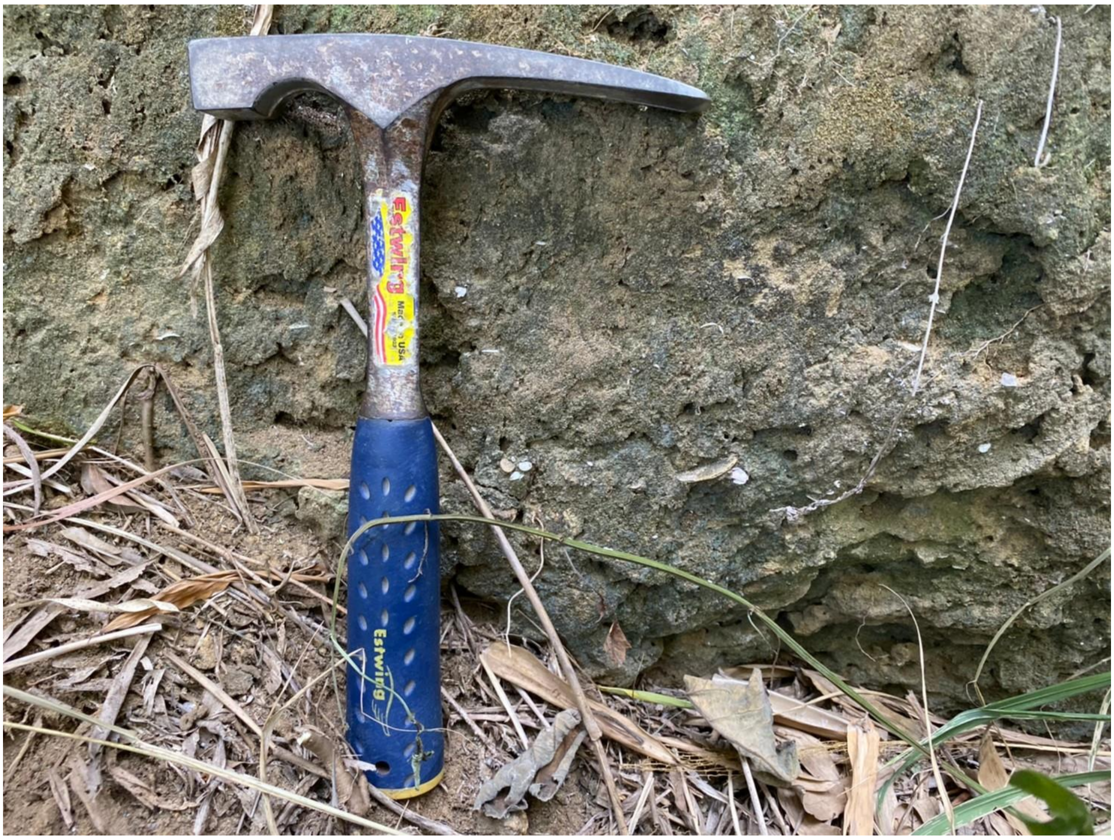

Fig. S1. Outcrop (site 1 on Fig. 2) photo of *Scaphechinus mirabilis* in Miaoli, Taiwan.

Identifier	$d^{13}C_{vpdb}$	$d^{18}O_{vpdb}$	Location	Type
M001	0.289	1.743	Japan	living
M002	0.319	1.922	Japan	living
M003	0.567	1.925	Japan	living
M005	0.150	1.909	Japan	living
M006	0.386	1.937	Japan	living
M007	0.768	1.732	Japan	living
M012	-1.487	1.908	Japan	living
M018	-0.356	0.983	Japan	living
M032	-0.031	0.929	Japan	living
M033	-0.227	0.845	Japan	living
M034	0.233	1.202	Japan	living
M035	-0.116	1.159	Japan	living
M036	-0.092	0.941	Japan	living
M037	-0.129	0.874	Japan	living
M038	-0.079	0.913	Japan	living
M039	-0.170	1.715	Japan	living
M040	0.029	1.475	Japan	living
M041	-0.156	1.055	Japan	living
T001	1.446	1.027	Taiwan	fossil
T002	-0.133	0.403	Taiwan	fossil
T003	-0.081	0.760	Taiwan	fossil
T004	-1.800	-0.391	Taiwan	fossil
T005	-0.991	-0.644	Taiwan	fossil
T006	0.084	0.177	Taiwan	fossil
T007	-1.460	0.572	Taiwan	fossil
T008	0.045	-0.408	Taiwan	fossil
T009	-0.657	0.206	Taiwan	fossil
T010	-0.559	-0.398	Taiwan	fossil
T013	0.346	0.631	Taiwan	fossil
T014	0.974	1.527	Taiwan	fossil
T015	0.242	0.510	Taiwan	fossil
M101A	1.027	0.427	Taiwan	fossil
M101C	1.290	0.673	Taiwan	fossil
SM002	0.573	1.417	Japan	living

Table S1. Stable carbon and oxygen isotopes derived from both living and fossil sand dollar *S. mirabilis* (see Fig. 3A). Japanese specimens were collected from Mutsu Bay by Satoshi Takeda. Taiwanese samples were collected from Site 2 in Fig. 2.

Easotope Name	M101A	M101C	SM002
Sample name	M101A	M101C	SM002
Mineralogy	Calcite	Calcite	Calcite
Nb replicates	3	3	3
d13C VPDB	1.027	1.290	0.573
d13C VPDB (SD)	0.133	0.078	0.025
d13C VPDB (SE)	0.077	0.045	0.015
d18O VPDB (Final)	0.427	0.673	1.417
d18O VPDB (SD)	0.261	0.136	0.112
d18O VPDB (SE)	0.151	0.079	0.064
D47 I-CDES	0.625	0.644	0.647
D47 I-CDES (SD)	0.008	0.004	0.026
D47 I-CDES (SE)	0.004	0.002	0.015
Temperature (˚C)	14	9	8
Tmin [+1SE]	13	8	4
Tmax [-1SE]	16	9	12

Table S2. Clumped oxygen isotopes derived from both living and fossil sand dollar *S. mirabilis* (see Fig. 3B).

Longitude	Latitude	Water Depth (m)	Temp. (°C)	SD Temp. (°C)
120.5	24.5	0	24.13	3.17
120.5	24.5	5	24.09	3.11
120.5	24.5	10	23.99	3.03
120.5	24.5	15	23.88	2.97
120.5	24.5	20	23.78	2.91
120.5	24.5	25	23.69	2.89
120.5	24.5	30	23.60	2.84
120.5	24.5	35	23.53	2.75
120.5	24.5	40	23.46	2.71
120.5	24.5	45	23.38	2.66
120.5	24.5	50	23.28	2.63
		**MEAN**	**23.71**	
		**SD**	**0.29**	

Table S3. World Ocean Atlas 2018 (annual mean) data of ocean temperature at depths.

Longitude	Latitude	Water Depth (m)	Temp. (°C)	SD Temp. (°C)
120.75	24.5	5	27.03	1.68
120.75	24.5	10	26.84	1.66
120.75	24.5	15	26.76	1.69
120.75	24.5	20	27.14	1.51
120.75	24.5	25	26.99	1.60
120.75	24.5	30	27.09	1.91
120.75	24.5	35	26.42	2.01
120.75	24.5	40	26.42	2.01
120.75	24.5	45	26.42	2.01
120.75	24.5	50	26.41	2.01
		**MEAN**	**26.75**	
		**SD**	**0.31**	

Table S4. Ocean temperature at depths retrieved from Ocean Data Bank of the Ministry of Science and Technology, Republic of China (http://www.odb.ntu.edu.tw/).
